# Lipid metabolism during pregnancy: consequences for mother and child

**DOI:** 10.1097/MOL.0000000000000927

**Published:** 2024-02-21

**Authors:** Janneke W.C.M. Mulder, D. Meeike Kusters, Jeanine E. Roeters van Lennep, Barbara A. Hutten

**Affiliations:** a Department of Internal Medicine, Erasmus MC Cardiovascular Institute, University Medical Center Rotterdam, Rotterdam; b Department of Pediatrics; c Department of Epidemiology and Data Science, Amsterdam University Medical Center, University of Amsterdam; d Amsterdam Cardiovascular Sciences Research Institute, Diabetes & Metabolism, Amsterdam, The Netherlands

**Keywords:** cardiovascular risk, familial hypercholesterolemia, lipids, pregnancy

## Abstract

**Purpose of review:**

Accommodating fetal growth and development, women undergo multiple physiological changes during pregnancy. In recent years, several studies contributed to the accumulating evidence about the impact of gestational hyperlipidemia on cardiovascular risk for mother and child. This review aims to provide a comprehensive overview of the current research on lipid profile alterations during pregnancy and its associated (cardiovascular) outcomes for mother and child from a clinical perspective.

**Recent findings:**

In a normal pregnancy, total and LDL-cholesterol levels increase by approximately 30–50%, HDL-cholesterol by 20–40%, and triglycerides by 50–100%. In some women, for example, with familial hypercholesterolemia (FH), a more atherogenic lipid profile is observed. Dyslipidemia during pregnancy is found to be associated with adverse (cardiovascular) outcomes for the mother (e.g. preeclampsia, gestational diabetes, metabolic syndrome, unfavorable lipid profile) and for the child (e.g. preterm birth, large for gestational age, preatherosclerotic lesions, unfavorable lipid profile).

**Summary:**

The lipid profile of women during pregnancy provides a unique window of opportunity into the potential future cardiovascular risk for mother and child. Better knowledge about adverse outcomes and specific risk groups could lead to better risk assessment and earlier cardiovascular prevention. Future research should investigate implementation of gestational screening possibilities.

## Graphical abstract

**Figure:**
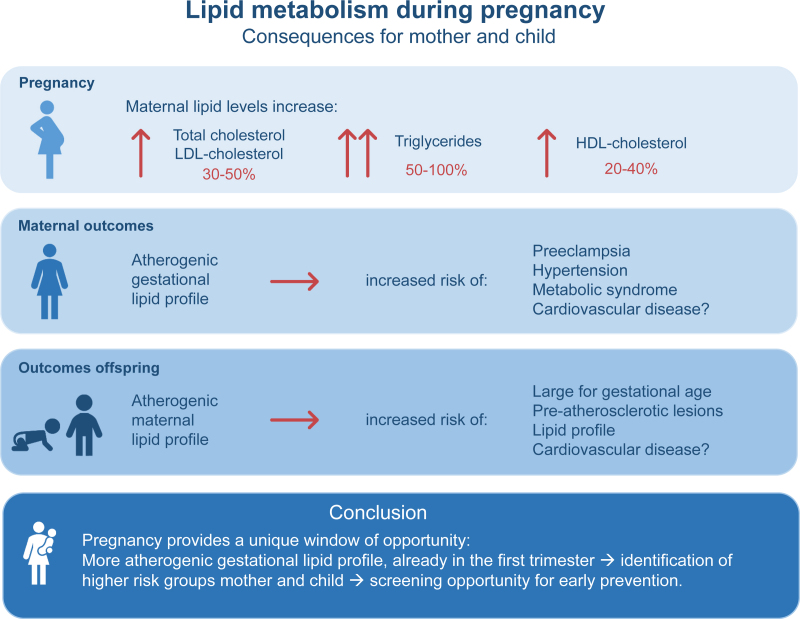

## INTRODUCTION

During pregnancy, the maternal metabolism undergoes adaptations to accommodate the growth and development of the fetus. With regards to the lipoprotein metabolism, these changes consist of elevated lipids in gestating women, including low-density lipoprotein cholesterol (LDL-C). Elevated LDL-C is an important causal risk factor for atherosclerotic cardiovascular disease (ASCVD) [1]. The ASCVD risk accumulates with increasing LDL-C concentration and a longer duration of exposure [1].

Periods in life with elevated LDL-C levels, such as pregnancy, contribute to the overall life-time cholesterol burden. In recent years, several studies have reported on the association between the atherogenic lipid profile during pregnancy and adverse short and long-term outcomes for both the mother and the child [2,3^▪▪^,4^▪^,^5^–^7^]. An increased risk of developing an atherogenic lipid profile during pregnancy has been observed in specific risk groups such as women with familial hypercholesterolemia (FH) [8].

This review will summarize changes in maternal lipid profile during pregnancy, and the impact of gestational dyslipidemia on short and long-term outcomes for the mother child. The focus of this review will be on women from the general population and women with FH. 

**Box 1:**
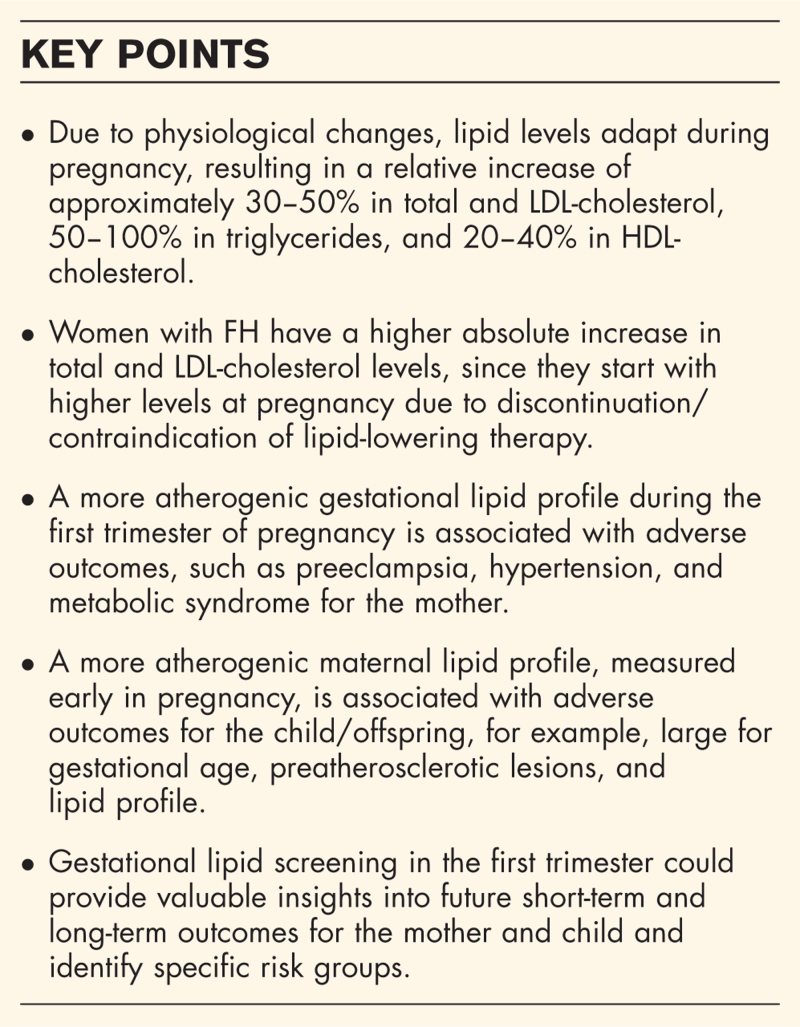
no caption available

## LIPID PROFILES DURING PREGNANCY

During pregnancy, physiological changes occur in the maternal lipid metabolism which are necessary for the preservation of pregnancy and fetal development and growth. Triglycerides are apart from glucose the major sources for fetal energy. Cholesterol is essential for embryonic and fetal development, as it is an indispensable component of the cell membranes as well as of lipid-rafts which are responsible for numerous intracellular signaling functions. Moreover, an increase of cholesterol is needed to meet the increased demand for maternal and placental steroids. Maternal lipoproteins do not cross the placenta but bind the specific receptors on the syncytiotrophoblast of the placental villi [9]. Cholesterol and triglycerides are transported through these cells to the fetal circulation [9]. In contrast to the lipid metabolism in adults, fetal HDL, which has a different composition compared to adult HDL, is the main cholesterol carrier [10].

### Women from the general population

In the first and second trimester, the body is in an anabolic state to prepare for the fetal energy requirements later on in pregnancy. While in the last trimester, the lipid metabolism changes to the catabolic phase with a decline of fat accumulation [11]. This phase is characterized by increased lipolysis and mobilization of triglycerides from adipocytes. Increased production of very LDL (VLDL) in combination with impaired lipoprotein lipase (LPL) activity leads to inefficient clearance of triglyceride-rich lipoproteins (TRLs) such as VLDL and VLDL-remnants, resulting in increased triglycerides levels [11].

**FIGURE 1:**
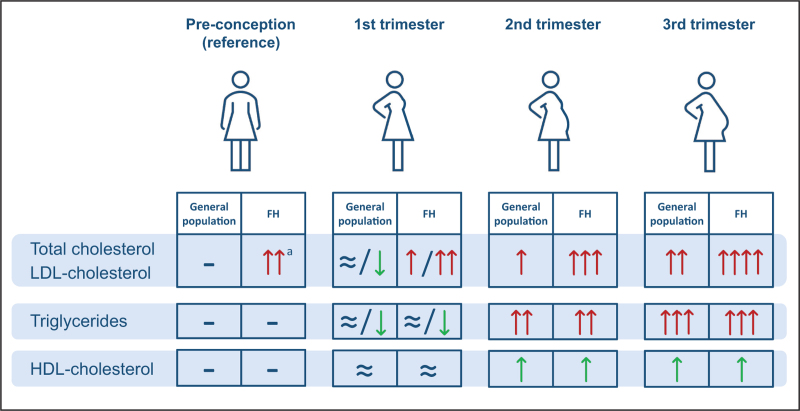
Relative changes in lipids during preconception (reference) and pregnancy. ^a^ During an active pregnancy wish, women with familial hypercholesterolemia (FH) are currently advised to discontinue lipid-lowering therapy. In pregnancy, women with and without FH undergo physiological changes in lipid metabolism. This results in comparable relative changes in lipid levels [8]. However, the absolute increase in mmol/l (or mg/dl) in women with FH will be larger since they start pregnancy already with a higher total and LDL-cholesterol level prior to pregnancy.

The aforementioned processes lead to trimester-dependent changes in lipid levels during pregnancy (Fig. 1). In early pregnancy, total cholesterol, LDL-C, triglycerides, and apolipoprotein B (apoB) levels decrease slightly and increase from the second trimester until end of term [12]. Total cholesterol and LDL-C levels increase approximately 30–50% while triglycerides increase about 50–100% [13^▪▪^]. HDL-C levels and apolipoprotein A1 (apoA1) increase 20–40% from early pregnancy onwards and plateau around 20–24 weeks [12]. Lipid levels in pregnancy and the magnitude of these changes during pregnancy are influenced by many factors, including prepregnancy lipid levels and BMI, age, diet, and ethnicity [8,14,15^▪^,^16^,^17^].

### Women with familial hypercholesterolemia

FH is a prevalent (∼1 : 313) autosomal semi-dominant condition caused by mutations in genes involved in the lipid metabolism, resulting in elevated LDL-C levels and premature ASCVD risk [18,19]. Women with heterozygous FH have total cholesterol and LDL-C levels which are approximately twice as high compared to women without FH. Results from a Norwegian study showed that, although the relative increase in total cholesterol and LDL-C levels between 17–20 and 36 weeks’ gestation is similar in women with FH compared to those without FH (28.7 vs. 25.4% and 29.6 vs. 34.2%, respectively; Fig. 1), the absolute increase is much higher in women with FH leading to higher absolute increases in lipid levels during pregnancy (e.g. LDL-C increase of approximately +1.9 compared to +0.8 mmol/l, respectively) [8]. Although in the normal range, triglyceride levels were also higher in women with FH compared to women without FH and showed a similar relative increase (116 vs. 103%), while HDL-C did not increase in either women with or without FH [8].

Women with homozygous FH have extremely elevated total cholesterol and LDL-C levels and an extreme risk of very premature cardiovascular disease. Case reports of pregnancies in women with homozygous FH have been described [20^▪^]. Because of the even higher baseline lipid levels, women with homozygous FH have even more pronounced increases in total cholesterol and LDL-C levels, although most are treated with lipoprotein apheresis and sometimes statins and ezetimibe even though these are contraindicated in pregnancy [20^▪^].

## DYSLIPIDEMIA IN PREGNANCY: CONSEQUENCES FOR THE MOTHER

### Mothers from the general population

It has been shown that an atherogenic lipid profile increases the risk of endothelial damage through oxidative stress mechanisms in the arterial vessel wall. In pregnant women, this could possibly lead to gestational (pre)hypertension and may result in sustained hypertension postpartum.

In a large (*n* = 5690 women) ongoing population-based prospective birth cohort, maternal lipid profile was determined in early pregnancy [2]. Blood pressure was measured in early, mid, and late pregnancy, and 6 and 9 years after pregnancy. Early gestational lipid levels were not associated with gestational hypertension, while total cholesterol, LDL-C, non-HDL-C, and especially triglycerides were positively associated with blood pressure in pregnancy and at 6 and 9 years after pregnancy [2]. Moreover, triglycerides were positively associated with sustained hypertension 6 and 9 years after pregnancy. The authors concluded that lipid levels in early pregnancy were associated with a cardiovascular burden for the mother by increasing the risk of preeclampsia and sustained hypertension, and may therefore serve as an early marker for later-life cardiovascular disease. A recent study with 12 715 Chinese women reported similar results for triglycerides: elevated triglycerides in early pregnancy were associated with preeclampsia [adjusted odds ratio (OR) 1.75; 95% confidence interval (95% CI) 1.29–2.36] and gestational diabetes mellitus (GDM) (adjusted OR 1.95; 95% CI 1.69–2.25) [3^▪▪^]. In a retrospective study of 881 women with a twin pregnancy, the significant association between first trimester triglycerides and preeclampsia and gestational diabetes was also observed [21]. A specific feature of dyslipidemia in pregnancy is increased HDL-C, which could possibly lead to maternal endothelium protection. In a meta-analysis, it was shown that HDL-C might play a protective role in developing preeclampsia [4^▪^].

A particular group of interest are women with GDM. GDM defined as glucose intolerance leading to hyperglycemia occurs in women without known diabetes, usually in the second term of pregnancy. Women who will develop GDM have a distinct metabolomic profile hallmarked by smaller HDL than women who will not develop GDM [22]. Compared to women without GDM, women with GDM have higher total cholesterol and especially higher triglycerides levels in the second and third trimester [23^▪^].

A recent Chinese study developed a machine-learning based prediction tool to diagnose GDM in early pregnancy [24^▪▪^]. Interestingly, in the optimal 7-variable model, triglycerides level was included in addition to established risk factors such as age, family history or previous GDM, and glucose metabolism variables. In conclusion, women with GDM have a distinct adverse lipid profile in pregnancy, possibly early gestational lipid levels can be used for early detection of GDM.

In a prospective population-based cohort study (3510 women), the association between gestational lipid levels (at median 13.2 weeks) and lipid levels and prevalence of the metabolic syndrome 6 years after pregnancy was studied [5]. Gestational lipid levels were positively associated with corresponding lipid levels 6 years after pregnancy, independent of pregnancy complications. Compared to women without metabolic syndrome, women with metabolic syndrome 6 years after pregnancy had a more atherogenic lipid profile in early pregnancy, which was significant for all analyzed lipids. Gestational triglycerides in the highest quartile and HDL-C in the lowest quartile were associated with the highest risk for future MS, independent of smoking and BMI. It was therefore concluded that gestational lipid levels provide an insight in the future cardiovascular risk profile of women in later life. Monitoring and lifestyle intervention could be indicated in women with an unfavorable gestational lipid profile to optimize timely cardiovascular risk prevention. Studies have shown increased risk of ASCVD with higher parity [25,26^▪^], such as coronary heart disease [27,28] and carotid plaque presence and progression [29^▪▪^]. As there are several physiological changes during pregnancy, future research should further investigate the impact of gestational lipid levels on short-term and long-term outcomes. Specifically, further knowledge is needed about the (repetitive) impact of a more atherogenic gestational lipid profile on future cardiovascular risk.

### Mothers with familial hypercholesterolemia

**FIGURE 2:**
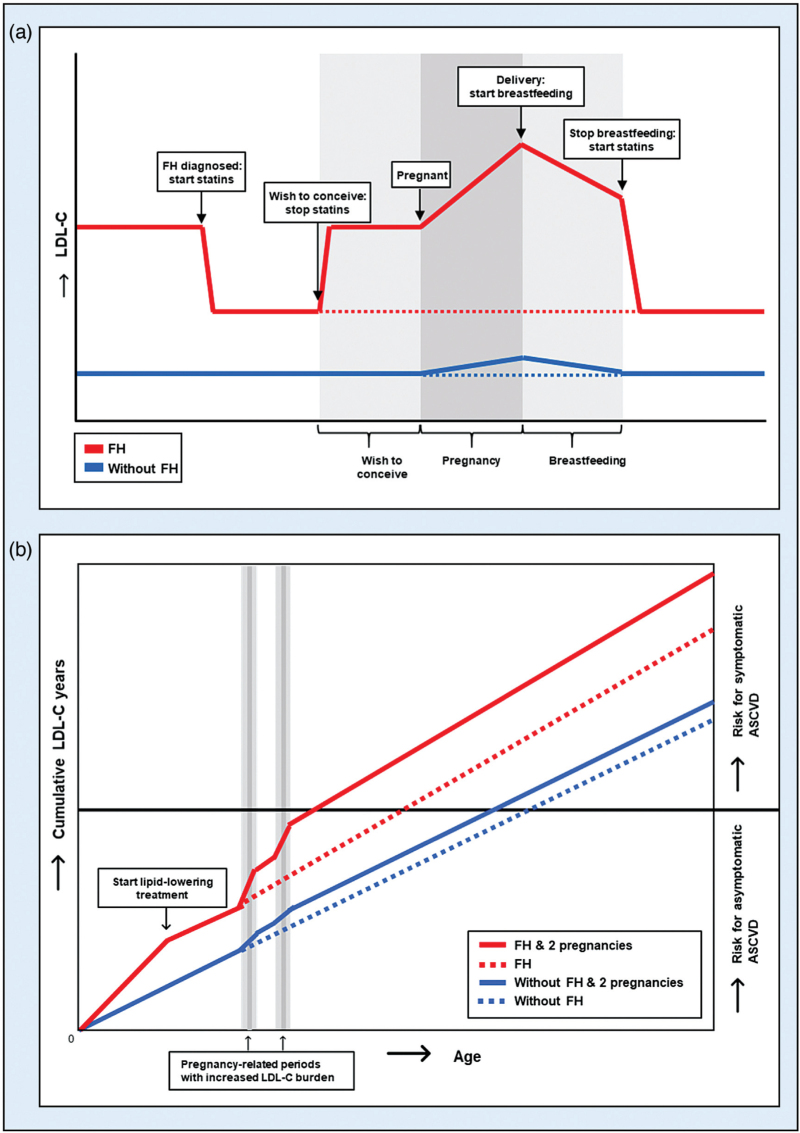
(a) Low-density lipoprotein cholesterol levels during pregnancy in women with and without FH. (b) Schematic representation of possible impact of gestational lipid profile on cholesterol burden in women with and without familial hypercholesterolemia.

Several studies indicate that a (transient) atherogenic lipid profile during pregnancy is associated with an increased adverse maternal ASCVD risk profile (short-term and long-term) [30]. It can be hypothesized that in women with FH the risk for adverse outcomes will be higher, due to their already higher levels of total cholesterol, LDL-C, and triglycerides, and in which the physiological rise during pregnancy is more pronounced [8]. In addition, it is further magnified in women having more than one pregnancy (Fig. 2) and amplified by the discontinuation of cholesterol-lowering therapy. The latter often spans a period much longer than pregnancy itself, since women with FH are advised to discontinue lipid-lowering drugs already when they consider pregnancy up to and including breast-feeding the newborn.

Results of a recent study on the duration of pregnancy-related off-statin periods including the breastfeeding period in 102 women with FH showed a median (min-max) total length of pregnancy-related off-statin periods of 2.3 (0–14.2) years [31^▪^]. Lost statin median (min-max) treatment time was 18 (0–100)% at mean (SD) age of 31 (4.3) years at last pregnancy. These findings indicate that young women with FH lose years of treatment when discontinuing statins in relation to pregnancy and breastfeeding periods. The authors conclude that these women should be closely followed up to minimize the duration of these off-statin periods. Whether these periods of interrupted treatment increase the cardiovascular risk in FH women needs to be elucidated.

Little is known about the impact of pregnancy on cardiovascular outcomes in women with homozygous FH and this deserves further investigation.

### Screening of future mothers

Since studies have shown that already early gestational lipid levels are associated with short- and long-term outcomes for the mother (Table 1), the first trimester could be an opportune moment to perform gestational lipid screening. A recent American study with 445 patients has shown that gestational lipid screening is feasible and observed abnormal lipid levels in 25% of the screened women. One patient with previously undiagnosed suspected FH was identified [32^▪^]. In the Netherlands, gestational lipid screening could, for example, be added to the current screening program for HIV, syphilis, hepatitis B virus, and red-blood-cell immunization, that is offered to all pregnant women in the first trimester. This screening program achieved a coverage of more than 99% of all Dutch pregnant women over the last years (2005–2021) [33,34].

**Table 1:** Short-term and long-term outcomes that were found to be associated with a more atherogenic lipid profile during pregnancy for the mother and offspring

	Mother	Child
Short-term^a^	Preeclampsia [2,3^▪▪^,4^▪^] Gestational diabetes [3^▪▪^,^22^,^24^^▪▪^]	Preterm delivery^b^[3^▪▪^,^39^–^41^] Large for gestational age [3^▪▪^,^41^] Preatherosclerotic lesions [35–37]
Long-term	Hypertension [2] Metabolic syndrome [5] Lipid profile [5]	Lipid profile [6,42,43^▪▪^]

a Short-term outcomes during pregnancy or the first year postpartum.

b Significant in most studies with the exception of one study [38].

## DYSLIPIDEMIA DURING PREGNANCY: CONSEQUENCES FOR THE OFFSPRING

### Offspring of mothers from the general population

In autopsy studies in spontaneously aborted fetuses, offspring from mothers with hypercholesterolemia exhibited significantly more and larger preatherosclerotic lesions of the aorta than offspring from mothers with normal cholesterol levels [35,36]. In addition, more progression of preatherosclerotic lesions of the aorta in offspring from hypercholesterolemic mother was seen in another autopsy study [37].

Hyperlipidemia has been considered an instigator of inflammation and oxidative stress and suggested to be associated with adverse pregnancy outcomes including preterm delivery. In a recent multicenter cohort study, this association was evaluated in 239 pregnant women aged 20–35 years [38]. No statistically significant difference in spontaneous preterm delivery between pregnant women with and without hyperlipidemia was found, but they did not adjust for possible confounders. In two systematic reviews, it was suggested that maternal dyslipidemia during pregnancy, either elevated total cholesterol or triglycerides, was associated with an increased risk of preterm birth [39,40]. In addition, a Chinese study with 12 715 women, observed that elevated triglycerides in early pregnancy were associated with preterm delivery, similar to findings in the Dutch Amsterdam Born Children and their Development (ABCD) study [3^▪▪^,^41^]. Both studies also reported an association for elevated maternal triglyceride levels measured during early pregnancy and children being born large for gestational age.

It was suggested that increased cholesterol levels during pregnancy are associated with overweight in offspring, but this was not confirmed in the ABCD cohort in children at age 11–12 years [15^▪^]. Results from this same cohort further showed that maternal early gestational lipid profile is associated with offspring's lipid profile at age 5–6 years [6]. Similar to findings from a Norwegian study in 61 mother-children pairs [42], in the ongoing population-based Generation R study, lipid profile in early pregnancy (*n* = 2692, median 13.2 weeks) was associated with the lipid profile of children at 6 and 10 years after pregnancy [43^▪▪^], independent of maternal BMI and diet.

Recently, an Italian retrospective study in 89 patients with an acute myocardial infarction and 221 controls observed an association between maternal gestational cholesterol levels in the first and second trimester and adult BMI and severity of myocardial infarction in offspring [7].

### Offspring of mothers with familial hypercholesterolemia

The population of patients with FH offers a unique opportunity to further study the impact of exposure to hypercholesterolemia *in utero*. As hypercholesterolemia in patients with FH is explained by the presence of a single gene variation, the hypothesis that gestational hypercholesterolemia affects the offspring, can be well explored by comparing offspring from FH mothers (who were exposed to high cholesterol levels *in utero*) with offspring from FH fathers (who were not exposed to elevated cholesterol levels *in utero*). Although one study suggested that cholesterol levels were slightly higher in adult offspring from FH mothers as compared to adult offspring from FH fathers [44], this could not be confirmed in other studies. A large study of individuals including both children and adults did not show a more atherogenic lipid profile or greater carotid intima-media thickness, a marker of atherosclerosis, in offspring from mothers with FH than offspring from fathers with FH. This was the case for both offspring with FH and offspring who did not inherit FH [45]. A study with 1063 Norwegian and Dutch children with FH also found no significant differences in lipid levels when stratifying by parental inheritance [46]. Furthermore, recent data from Spain showed no differences in lipid levels in adult offspring from mothers with FH compared to adult offspring from fathers with FH nor in the sex and age adjusted comparison of cardiovascular disease prevalence (9.2 vs. 9.3%) [47^▪^]. Therefore, these studies do not support the possible mechanism of epigenetic programming of metabolism during fetal development as a result of higher cholesterol exposure *in utero*.

On the other hand, a significant association between maternal inheritance and increased coronary artery calcium scores was observed in 1350 French patients, but no difference in prevalence of cardiovascular events [48^▪^]. Surprisingly, a Canadian study found a younger age at first event and a 1.5-fold increased risk of ASCVD adjusted for confounders with paternal inheritance compared to maternal inheritance [49]. More studies in FH are needed to elucidate the contradictory findings regarding inheritance pattern and lipid levels and cardiovascular outcomes.

## CONCLUSION

During pregnancy, there are substantial changes in the lipid metabolism of women, such as increases in total cholesterol, LDL-C, and triglycerides from the second to third trimester. Studies of mostly observational nature have shown associations between a more atherogenic lipid profile during pregnancy and adverse outcomes later on in life. In addition, studies suggest that gestational lipid levels are associated with adverse pregnancy outcomes, and both lipid levels and cardiovascular risk in offspring. As observed in studies in offspring from parents with FH, this phenomenon cannot be explained to a high cholesterol exposure during pregnancy alone. Possibly, other (genetic) factors play a role.

A special group of concern are woman with FH. Because of their already higher levels of cholesterol and discontinuation of statin therapy for a period that is much longer than during pregnancy alone, pregnancy is likely a vulnerable time for ASCVD risk progression. Therefore, these women need to be closely monitored during pregnancy.

The pregnancy period could provide a unique window of opportunity to identify women with a more atherogenic lipid profile who are at a higher cardiovascular risk, and lipid levels in pregnancy could be used as early predictors of the long-term cardiovascular health of the offspring. Screening of these gestational lipid levels could expose the need for early interventions to decrease the mother's and offspring's lipid levels and reduce their cardiovascular risk later in life. In addition, it could play an important role in timely diagnosis and management of women with FH, followed by cascade screening in order to possibly diagnose first-degree family members. Screening of gestational lipid levels could be combined with current gestational screening programs, for example, for HIV, during the first trimester. Future research should further investigate the specific women at increased risk and possible benefits of implementing gestational screening in early pregnancy and risk prevention management for mother and child.

## Acknowledgements

*None.*

### Financial support and sponsorship

*None.*

### Conflicts of interest

*J.M. and D.M.K. report none. J.R.V.L. received a research grant from Novartis paid to the institution. B.A.H. received a research grant from Silence Therapeutics paid to the institution.*

## REFERENCES AND RECOMMENDED READING

Papers of particular interest, published within the annual period of review, have been highlighted as:

▪ of special interest
▪▪ of outstanding interest
